
JOURNAL INFORMATION
==============================
NLM Title Abbreviation: ScientificWorldJournal
Journal ID: ScientificWorldJournal
NLM Journal ID: 101131163
Journal ID: TSWJ

The Scientific World Journal

ISSN: 2356-6140
EISSN: 1537-744X
Publisher: Wiley

ARTICLE INFORMATION
==============================
PMCID: PMC5901328
PMID: 18167618
DOI: 10.1100/tsw.2007.299
Article ID: 794098
Article version: 1
Subjects: Discussion Paper

Construction of Efficacious Gait and Upper Limb Functional Interventions Based on Brain Plasticity Evidence and Model-Based Measures For Stroke Patients

Daly Janis J. 1 2 *
Ruff Robert L. 3
1 Stroke Motor Control and Motor Learning Laboratory, DVA FES Center of Excellence, Louis Stokes Cleveland VA Medical Center, USA
2 Department of Neurology, Case Western Reserve University School of Medicine, Cleveland, USA
3 National Director of Neurology, Veterans Affairs Central Office and Medical Director, Cleveland FES Center, USA
*Janis J. Daly: jjd17@case.eduAcademic Editor: Robert B. Daroff

Publication date: 2007
Electronic publication date: 2007 Dec 20
Volume: 7
First page: 2031
Last page: 2045
Received 2007 Feb 5; Revised 2007 Oct 19; Accepted 2007 Oct 22
Copyright: Copyright © 2007 Janis J. Daly and Robert L. Ruff.
Copyright year: 2007
License: This is an open access article distributed under the Creative Commons Attribution License, which permits unrestricted use, distribution, and reproduction in any medium, provided the original work is properly cited.
License URL: https://creativecommons.org/licenses/by/3.0/

Keywords: stroke, CVA, gait, upper limb function, mobility, robotics, treadmill training, body weight supported treadmill training, gait training, motor learning, functional electrical stimulation, FES

Funding: Rehabilitation Research and Development Service B2226R Rehabilitation Research and Development Service A3102R

==============================
Discussion Paper
*TheScientificWorldJOURNAL* (2007) 7, 2031–2045
ISSN 1537-744X; DOI 10.1100/tsw.2007.299

TheScientificWorldJOURNAL
www.thescientificworld.com

### Construction of Efficacious Gait and Upper Limb Functional Interventions Based on Brain Plasticity Evidence and Model-Based Measures For Stroke Patients

Janis J. Daly[1,2,]* and Robert L. Ruff[3]

*[1]Director, Stroke Motor Control and Motor Learning Laboratory and Associate Director, DVA FES Center of Excellence, Louis Stokes Cleveland VA Medical Center; [2]Department of Neurology, Case Western Reserve University School of Medicine, Cleveland; [3]National Director of Neurology, Veterans Affairs Central Office and Medical Director, Cleveland FES Center*

**E-mail**: jjd17@case.edu; Robert.Ruff@med.va.gov

*Received February 5, 2007; Revised October 19, 2007; Accepted October 22, 2007; Published December 20, 2007*

---

**For neurorehabilitation to advance from art to science, it must become evidence-based. Historically, there has been a dearth of evidence from which to construct rehabilitation interventions that are properly framed, accurately targeted, and credibly measured. In many instances, evidence of treatment response has not been sufficiently robust to demonstrate a change in function that is clinically, statistically, and economically important. Research evidence of activity-dependent central nervous system (CNS) plasticity and the requisite motor learning principles can be used to construct an efficacious motor recovery intervention. Brain plasticity after stroke refers to the regeneration of brain neuronal structures and/or reorganization of the function of neurons. Not only can CNS structure and function change in response to injury, but also, the changes may be modified by "activity". For gait training or upper limb functional training for stroke survivors, the "activity" is motor behavior, including coordination and strengthening exercise and functional training that comprise motor learning. Critical principles of motor learning required for CNS activity-dependent plasticity include: close-to-normal movements, muscle activation driving practice of movement; focused attention, repetition of desired movements, and training specificity.**

**The ultimate goal of rehabilitation is to restore function so that a satisfying quality of life can be experienced. Accurate measurement of dysfunction and its underlying impairments are critical to the development of accurately targeted interventions that are sufficiently robust to produce gains, not only in function, but also in quality of life. The Classification of Functioning, Disability, and Health Model (ICF) model of disablement, put forth by the World Health Organization, can provide not only some guidance in measurement level selection, but also can serve as a guide to incorporate function and quality of life enhancement as the ultimate goals of rehabilitation interventions.**

**Based on the evidence and principles of activity-dependent plasticity and motor learning, we developed gait training and upper limb functional training protocols. Guided by the ICF model, we selected and developed measures with characteristics rendering them most likely to capture change in the targeted aspects of intervention, as well as measures having membership not only in the impairment, but also in the**

*Corresponding author.
©2007 with author.
Published by TheScientificWorld; www.thescientificworld.com

**functional or life role participation levels contained in the ICF model. We measured response to innovative gait training using a knee flexion coordination measure, coefficient of coordination consistency (ACC) of relative hip/knee (H/K) movement across multiple steps (H/K ACC), and milestones of participation in life role activities. We measured response to upper limb functional training according to measures designed to quantify functional gains in response to treatment targeted at wrist/hand or shoulder elbow training (Arm Motor Ability Test for wrist/hand (AMAT W/H) or shoulder/elbow (AMAT S/E)).**

**We found that there was a statistically significant advantage for adding FES-IM gait training to an otherwise comparable and comprehensive gait training, according to the following measures: H/K ACC, the measure of consistently executed hip/knee coordination during walking; a specific measure of isolated joint knee flexion coordination; and a measure of multiple coordinated gait components. Further, enhanced gains in gait component coordination were robust enough to result in achievement of milestones in participation in life role activities. In the upper limb functional training study, we found that robotics + motor learning (ROB ML; shoulder/elbow robotics practice plus motor learning) produced a statistically significant gain in AMAT S/E; whereas functional electrical stimulation + motor learning (FES ML) did not. We found that FES ML (wrist/hand FES plus motor learning) produced a statistically significant gain in AMAT W/H; whereas ROB ML did not. These results together, support the phenomenon of training specificity in that the most practiced joint movements improved in comparison to joint movements that were practiced at a lesser intensity and frequency. Both ROB ML and FES ML protocols addressed an array of impairments thought to underlie dysfunction.**

**If we are willing to adhere to the ICF model, we accept the challenge that the goal of rehabilitation is life role participation, with functional improvement as in important intermediary step. The ICF model suggests that we intervene at multiple lower levels (e.g., pathology and impairment) in order to improve the higher levels of function and life role participation. The ICF model also suggests that we measure at each level. Not only can we then understand response to treatment at each level, but also, we can begin to understand relationships between levels (e.g., impairment and function).**

**With the ICF model proffering the challenge of restoring life role participation, it then becomes important to design and test interventions that result in impairment gains sufficiently robust to be reflected in functional activities and further, in life role participation. Fortunately, CNS plasticity and associated motor learning principles can serve well as the basis for generating such interventions. These principles were useful in generating both efficacious gait training and efficacious upper limb functional training interventions. These principles led to the use of therapeutic agents (FES and robotics) so that close-to-normal movements could be practiced. These principles supported the use of specific therapeutic agents (BWSTT, FES, and robotics) so that sufficient movement repetition could be provided. These principles also supported incorporation of functional task practice and the demand of attention to task practice within the intervention. The ICF model provided the challenge to restore function and life role participation. The means to that end was provided by principles of CNS plasticity and motor learning.**

**KEYWORDS**: stroke, CVA, gait, upper limb function, mobility, robotics, treadmill training, body weight supported treadmill training, gait training, motor learning, functional electrical stimulation, FES.

### INTRODUCTION

For neurorehabilitation to advance from art to science, it must become evidence based. There is currently a dearth of evidence on which to construct rehabilitation interventions that are accurately targeted, properly framed, and credibly measured. In many instances, evidence of treatment response has not been sufficiently robust to demonstrate a change in function that is clinically, statistically, and economically important.

This paper describes how the evidence for activity-dependent central nervous system (CNS) plasticity, along with its motor learning principles, can be used to construct an efficacious intervention. Additionally, this paper provides an illustration of the use of a model to construct rehabilitation goals and to select an array of measurements of treatment response. The model is the World Health Organization (WHO) Disability Model, most recently referred to as the International Classification of Functioning, Disability, and Health Model (ICF[1,2,3]). We will describe measures developed specifically to quantify either coordinated gait components or functional performance of the upper limb.

### EVIDENCE FOR THE USE OF ACTIVITY-DEPENDENT CNS PLASTICITY AND MOTOR LEARNING PRINCIPLES TO CONSTRUCT MOTOR RECOVERY INTERVENTION

Activity-dependent CNS plasticity and the requisite motor learning principles can be used to construct an efficacious motor recovery intervention. Brain plasticity after stroke refers to the regeneration of brain neuronal structures and/or reorganization of the function of neurons. Not only can CNS structure and function change in response to injury, but the changes may also be modified by “activity”. During the past 15 years, researchers have provided us with a beginning understanding of the “activity” necessary to modulate CNS plasticity. In normal, mature animal models, an enriched environment, in comparison to a nonenriched environment, produced greater structural CNS changes, such as greater dendritic branching[4], greater number of synapses[5], and greater number of hippocampal neurons[6], as well as an association between CNS changes and motor behavioral gains[7,8]. The same activity-dependent plasticity was demonstrated in stroke animal models[4,9,10,11,12]. In human models, imaging studies provided preliminary evidence of activity-dependent plasticity[13,14,15,16,17,18,19,20,21].

For gait training or upper limb functional training for stroke survivors, the “activity” is motor behavior, including coordination and strengthening exercise, and functional training that comprises motor learning. Motor learning refers to the process of acquiring the capability to produce skilled movement[22]. The theory of motor learning is based on studies that span biomechanics, motor control, muscle biology, motor learning, and cognitive psychology[23]. A central assumption of the motor learning model used in neurorehabilitation is that the neural structures controlling movement are required to adapt to constraints that are imposed by the structure of the musculoskeletal system, the physical laws governing movement, and the impairments that are present[24]. Task-related practice of movement is considered essential[23]. Before generating the treatment plan, the therapist evaluates the point at which the patient fails at a given motor task. For example, the patient may not be able to bring a glass to the mouth without spilling the contents because there is an impairment of coordination for the combined movements of elbow flexion/forearm supination/wrist flexion/wrist lateral deviation, all of which are required in order to bring the glass to the mouth while keeping the surface of the liquid parallel to the ground (no spilling). Generation of the treatment plan involves determining a hierarchy of tasks or task components from less to greater difficulty[22,25]. In our example of “glass to the mouth without spilling”, depending on the coordination impairments that are present, one motor practice progression might be: elbow flexion + forearm supination; elbow flexion + wrist flexion; elbow flexion + wrist lateral deviation; and begin to combine three movements within a small range of motion, increasing the range as coordination is mastered; and begin to combine four of the required movements. In order to ensure successful response to intervention more certainly, some have studied the particular nature of the motor behavior practice necessary to effect activity-dependent CNS plasticity. The results of these studies provide us with several principles of motor learning required for CNS activity-dependent plasticity. These principles include close-to-normal movements[8,9], muscle activation driving practice of movement, focused attention[26], repetition of desired movements[27,28,29,30], and training specificity[31]. If an intervention includes practice of abnormal movements, unfocused attention, or too few repetitions, motor skill acquisition is unlikely to occur. In fact, one of the greatest difficulties for stroke survivors is the inability to produce volitional movements that are close to normal and that can be practiced repeatedly. Therefore, one of the critical hallmarks of an efficacious intervention is a method to practice close-to-normal movements repetitively, fulfilling the motor learning principles required to induce the brain plasticity needed to drive more normal movement.

### RATIONALE FOR THE USE OF THE ICF MODEL FOR MEASUREMENT SELECTION AND DEVELOPMENT

The ultimate goal of rehabilitation is to restore function so that an excellent quality of life can be experienced. However, for rehabilitation of many neurological diagnoses, credible evidence is lacking with regard to how to treat impairments sufficiently, so that function improves. Moreover, evidence is lacking regarding how to treat dysfunction sufficiently to actually improve quality of life. It appears that a health systems model could provide a means to focus the goal of rehabilitation on restoration of function and quality of life.

Accurate measurement of dysfunction and its underlying impairments is critical to the development of accurately targeted interventions that are sufficiently robust to produce gains not only in function, but also in quality of life. The ICF model of disablement, put forth by the World Health Organization, can provide some guidance in measurement level selection (ICF[1,2,3]). The ICF model provides a concept of the relationships among levels of abnormality for those with limitations. The ICF model includes components of pathology, impairment, dysfunction in performing personal tasks, and limitations in life role activities, arranged in a hierarchical fashion. The multiple components and the implied hierarchical configuration render the ICF model useful in identifying or developing the array of measures needed in order to accurately quantify response to rehabilitation. In the context of the ICF model, for stroke, impairment can include weakness or dyscoordination produced by nervous system pathology. In this model, dysfunction refers to loss or compromise of ability to perform an action or task basic to independent function, such as feeding oneself or walking to the bathroom. Limitation in life role activities refers to restrictions in performing social roles, such as employee, homemaker, spouse, family member, volunteer, or friend. The model suggests that we consider the pathologies and impairments that can underlie functional deficits[32]. For some patient populations, there is evidence that some dysfunctions are associated with particular underlying impairments[1]. The ICF model also suggests that dysfunction underlies limitations in life role activities.

Clearly, inaccurate measurement of treatment response is an obstacle when assessing the effectiveness of treatment. There are multiple measurement problems that preclude capture of an existing good effect size in response to treatment. First, the measure may have a ceiling or floor effect. For example, if stroke recovery was measured using the Barthel Index, 57.3% of patients were considered “recovered”, whereas if the NIH Stroke scale was used, only 44.9% of patients were considered recovered, and even fewer (36.8%) were recovered according to the Fugl-Meyer coordination scale[33]. Second, a measure may be insensitive at the level of intervention. This could be the case for the Functional Independence Measure (FIM[34,35]). For example, even if the intervention is targeted to enhance function of the involved upper limb, the FIM would likely not capture improved function of the involved limb. This could occur because the FIM measures gross overall function and does not distinguish whether a task is accomplished using one or both hands. Third, if the measure is too broad and includes irrelevant items that were not treated and so were not expected to change, a percent gain score may not capture change in response to treatment. Fourth, a problem in capturing gains can sometimes occur when all the relevant functional item scores are averaged rather than summed. For example, some measurement directions stipulate that functional tasks should be timed, the pre-/post-treatment gain calculated for each task, and then the mean across tasks calculated. However, a good improvement in one targeted functional task may be obliterated by averaging across the multiple items, some of which, although treated, showed no change. Fifth, some measures are scored using an item-weighting scheme based on assumptions that may be inaccurate for a given purpose. For example, the Craig Handicap Assessment Rating Tool[36] is an excellent measure of limitation in life role participation, with regard to the breadth and depth of item content and the response requirements from the user. However, if the intervention had a positive effect only on the items with lesser weights, it might not be possible to show a significant gain in response to treatment using the suggested weights. Sixth, some rehabilitation research measures are in a self-report format. A subject's viewpoint is useful to determine how patients feel about an intervention and how they perceive themselves, but self-report may not be an accurate measure of actual change in performance. Seventh, a measure may be heterogeneous. For example, a single measurement tool may contain items assessing both impairment and function (Wolf Motor Function Test[37]), or both gait deficits and compensatory strategies (Tinetti Gait Scale[38]). It is important to keep in mind both the limitations and advantages of each measure. But certainly, working from the ICF model, we can reason that if impairment is targeted in intervention, impairment should be measured. But importantly, if the ultimate goal of rehabilitation is normal function and life role participation, function and life role participation should also be measured. In that light and according to the ICF model, the intervention as a whole should be so structured and results robust enough to impact not only impairment, but also function and life role participation.

### EVIDENCE-BASED CONSTRUCTION OF A GAIT TRAINING INTERVENTION AND MODEL-BASED DEVELOPMENT OF MEASURES

### Intervention Development Based on Principles of Motor Learning and Brain Plasticity

Normal gait is characterized by multiple, simultaneously and/or sequentially executed coordinated and precisely timed movements of the hip, knee, and ankle that translate the body forward. Many stroke survivors exhibit persistent dyscoordinated gait components. A number of promising interventions have been studied and they include treadmill training (TT), body weight supported treadmill training (BWSTT), over ground gait training, and task-specific gait training. For the stance phase of gait, there is evidence that BWSTT can provide the *practice* of some gait components closer to normal than would otherwise be possible during volitional over ground gait training. These stance phase practice advantages include safety and balance control, mitigation of the fear of falling[39], more symmetrical single and double stance ratio[40], more symmetrical weight shift[39,41], more symmetrical activation of tibialis anterior and quadriceps during limb loading[42], and faster walking practice[39,43]. It is important to note that the prior list of items does not represent results of the treatment; rather, they are gait components that can be practiced with BWSTT.

In response to treatment, however, these methods have exhibited mixed results (e.g., BWSTT and TT[44]), according to measures of gait speed. In developing new gait training interventions, practicality is an important issue. In offering BWSTT, an abnormal swing phase can be practiced repetitively, unless normal swing phase movements are manually provided for each step. Manual assistance can be overly burdensome and is an unrealistic expectation for rehabilitation specialists. Studies of BWSTT and most gait training methods have either not measured coordinated gait components[44] or there was no improvement in coordinated gait components[45]. Possible reasons for the lack of results in some gait training studies could be that brain plasticity and associated motor learning principles were not sufficiently considered in construction of the intervention.

Our purpose was to develop a gait training protocol that would induce CNS plasticity that would be adequate to restore coordinated gait components to a sufficiently robust degree to impact participation in life role activities. According to the CNS plasticity model and associated motor learning principles, muscle contractions and movements can enhance CNS plasticity. This occurs, in part, through the affective signals, which are generated in muscle and joint receptors that are activated during movement and communicated to the CNS. The nature of the affective signals is important, requiring that the movements are properly performed as close to normal as possible and sufficiently repeated. In the case of more mildly involved stroke survivors, conventional exercise and gait training interventions can induce muscle contractions and movements. However, for many stroke survivors, the muscle contractions and movements are abnormal or nonexistent. There is a need to develop effective interventions for those stroke patients who are moderately or severely involved. Considering the evidence of CNS plasticity and associated principles of motor learning, we developed gait training methods that induced movement as close to normal as possible, and repeated practice of that movement.

FES (functional electrical stimulation) is a promising therapeutic modality in that it assists in satisfying CNS plasticity motor learning principles. Weakness[46] and dyscoordination[47] are impairments that can underlie gait deficits in stroke survivors. The literature supports the use of FES to address impairments of weakness[48,49], dyscoordination of single[50] and multiple joint[51] movements, abnormal muscle tone[52], and dyscoordinated gait components[51,50]. Requirements for an effective rehabilitation intervention include targeting the weakness and/or dyscoordination produced by a stroke, but also, importantly, effective rehabilitation must provide practice of functional movement components that are as close to normal as possible. FES can address several requirements of motor learning according to CNS plasticity. Using FES to drive muscle groups in specific activation patterns can provide strengthening exercise as well as retrain coordination. Coordination retraining can occur because FES can induce more normal movement patterns than could be practiced without FES. FES can be used in this way, even in some cases for which volitional movement practice is not possible at all. FES can be used to practice gait components separately and in over ground gait training.

In contrast to BWSTT that is not well suited for practice of swing phase gait components, there is evidence that FES can provide practice of more normal swing phase components[39]. Moreover, the combination of BWSTT and FES gait training can provide practice of a greater number of normal coordinated gait components for both stance and swing phases than either BWSTT or FES alone[39]. FES can induce muscle contractions and joint movements that can closely approximate normal gait components, as well as repeated practice of the gait components. Electrically induced muscle activations and swing phase movements are more actively produced in comparison with the passively received manual assistance of limb movements provided by therapists during BWSTT. FES can be customized to the needs of each patient with regard to stimulus intensity and timing of muscle activations within the gait cycle. We have found, for gait training, that FES delivered using implanted electrodes is superior to FES surface electrode systems. Multichannel surface FES systems are impractical for this purpose for a number of reasons, including a lengthy donning procedure, nonspecific muscle activation, inconsistent muscle response from day to day, impracticality of a home program (patients unable to don the system at home), and lack of portability of a multichannel surface system[53]. FES with intramuscular (IM) electrodes (FES-IM) can remedy these disadvantages. FES-IM proved safe[54], feasible for acute[55] and chronic[50] stroke survivors, comfortable for users[55], and capable of improving coordinated gait components[50,51]. In addition, with combination BWSTT + FES, we have the capability of practice of the greatest number of gait components for which we can satisfy two motor learning principles associated with CNS plasticity: practice of close-to-normal movement and repetition of that practice.

### Measurement Development

Coordinated gait components are required for normal walking[56,57]. We developed an FES-IM gait training protocol that targeted dyscoordination. Therefore, it was important to develop measures that were valid for that purpose and sensitive enough to discriminate gains that occurred in coordination in response to treatment. One movement that normally occurs during swing phase is knee flexion. The "stiff-legged gait" is one hallmark of a gait pattern exhibited by some stroke survivors[58]. In this pattern, the knee does not flex during swing phase. Volitional knee flexion is impaired in stroke survivors who utilize this stiff-legged pattern. To assess the potential improvement in response to our intervention, we devised a measure of knee flexion coordination impairment[59].

A second important aspect of the coordination of gait is the relative movement between hip and knee flexion during swing phase. Hip and knee flexion must occur according to consistently repeated, precise timing and precise excursion, with knee flexion occurring at a faster rate and to a greater degree than hip flexion. Without this precise timing and movement control, there is an abnormal elevation of energy cost of the gait pattern. Consistency of movement timing and excursion is a hallmark of normal gait[60]. The FES-IM intervention was developed to deliver consistent practice of these precision movement timings. Since we targeted consistent gait movement timing in our interventions, it was important to measure consistency of coordinated gait hip/knee movements. There was no existing single-number index for stroke gait coordination according to consistency of hip/knee coordination; therefore, we tested a potential measure for its usefulness in stroke gait evaluation, finding it reliable and capable of discriminating normal gait coordination from dyscoordination in stroke gait for both the involved and uninvolved limbs, as well as discriminating treatment response[60]. This measure was termed the average coefficient of consistency (ACC) of relative hip/knee (H/K) movement across multiple steps (H/K ACC).

### Results of Implementing an Evidence-Based Intervention with Model-Based Measures

In a randomized controlled trial (RCT; n = 31 stroke survivors >12 months poststroke), we tested comprehensive gait training utilizing BWSTT, both with and without FES-IM. This combination protocol was developed not only to address weakness and dyscoordination of isolated joint movement, but also to provide practice of stance phase and swing phase gait components that were executed as close to normal as possible. Table 1 lists an accounting of practice advantages of BWSTT, FES-IM, and a combination of the two. For the FES-IM group, the following muscles were provided with intramuscular electrodes: gluteus medius, vastus lateralis, short head of the biceps femoris, long head of the biceps femoris, either the semitendonosus or the semimenbrandosus, lateral head of the gastrocnemius, tibialis anterior, and peroneus longus. Monitored anesthesia care was provided for patient comfort. Electrodes were inserted using a hypodermic needle injection method[61] and electrodes remained comfortably in place for the duration of the study[54,62]. Subjects in both groups were treated 4 days/week, 1.5 h/day, for 12 weeks, with a single session composed of 0.5-h exercise, 0.5-h BWSTT, and 0.5-h over ground gait training. The FES-IM group used FES-IM for all three aspects of treatment and the No-FES did not use FES during the three aspects of treatment. FES gait training patterns were customized from templates[39,51]. FES-IM was used for strengthening and coordination exercise, gait component practice, and gait training, with the FES level incrementally reduced as volitional control was restored. Stimulation parameters were as follows: 30 Hz, 20 mA, 1- to 150-µsec pulse width. In the RCT, we found that there was a statistically significant advantage for adding FES-IM gait training, according to H/K ACC, the measure of consistently executed hip/knee coordination during walking[60] and a specific measure of isolated joint knee flexion coordination, and a measure of multiple coordinated gait components[59]. Further, enhanced gains in gait component coordination were robust enough to result in achievement of milestones in participation in life role activities[59]. One reason that these results were obtained could have been that the intervention was designed to provide practice of as-close-to-normal movement as possible, thereby satisfying one of the motor learning principles of CNS brain plasticity. Further, specific measures were used for impairments that were targeted in treatment: isolated joint movement coordination and coordinated gait components. Finally, guided by the health systems ICF model, life role participation was considered an important outcome. Therefore, the protocol was constructed so that there was incrementally graded progression of motor coordination and sufficient practice to restore function and life role participation.

**TABLE 1**
**Capability of Three Different Modalities to Provide Gait Practice Characteristics**

| **Gait Practice Characteristics** | **FES-IM Alone (No. of Subjects)** | **BWSTT Alone (No. of Subjects)** | **Combo FES-IM + BWSTT (No. of Subjects)** |
|---|---|---|---|
| Control of contralateral pelvic drop* | Partially achieved** (7/8[A]) | Partially achieved** (7/8[A]) | Yes*** (7/8[A]) |
| Eliminate excessive stance phase knee flexion or extension* | | | |
| Loading | Partially achieved** (7/8[A]) | Partially achieved** (7/8[A]) | Yes*** (7/8) |
| Mid stance | Partially achieved** (7/8[A]) | Partially achieved** (7/8[A]) | Yes*** (7/8[A]) |
| Terminal stance | No (0/8) | Partially achieved** (8/8) | Yes*** (8/8) |
| Eliminate excessive stance phase ankle inversion* | Partially achieved** (5/8[A,B]) | Partially achieved** (5/8[A,B]) | Yes*** (5/8[A,B]) |
| Eliminate impaired swing phase hip flexion excursion* | Partially achieved** (8/8[C]) | No (0/8) | Partially achieved** (8/8) |
| Eliminate impaired swing phase knee flexion excursion* | Yes*** (8/8[C]) | No (0/8) | Yes (8/8) |
| Eliminate excessive mid-swing ankle plantarflexion* | Yes*** (6/8[A,C]) | No (0/6[D]) | Yes*** (6/8[B]) |
| Facilitate greater walk speed | No (0/7[E]) | Yes*** (7/8[A]) | Yes*** (7/8[A]) |
| Prevent falls | No (0/8) | Yes*** (8/8) | Yes*** (8/8) |

* After the Rivermeade Gait Assessment (RGA) (Lord SE, Halligan PW, Wade DT. (1998) Visual gait analysis: the development of a clinical assessment and scale. Clin. Rehabil. **12(2)**,107-119)

** Partially achieved = at least one point greater on the RGA compared with the RGA score during over ground walking.

******* Yes = normal gait component achieved for practice.

[A] Remaining subjects demonstrated close-to-normal performance without modalities.

[B] The five listed subjects required an ankle brace (Air splint) for medial/lateral stability.

[C] With FES-IM driven muscle strength at least 3+; and the relevant (hip, knee, and ankle) Fugl-Meyer item score ≥1 (during use of FES-IM).

[D] Two subjects had normal ankle flexion prior to treatment; the remaining six subjects had no change in ankle flexion practice pattern with BWSTT.

[E] One subject had normal walking speed prior to treatment; the remaining seven had no change in walking practice speed with FES-IM. From Daly et al. (2004) *J. Neurol. Sci.*, Table 5.

### EVIDENCE-BASED CONSTRUCTION OF AN UPPER LIMB MOTOR LEARNING INTERVENTION AND MODEL-BASED DEVELOPMENT OF MEASURES

### Intervention Development Based on Principles of Motor Learning and Brain Plasticity

A number of innovative methods have been studied during the past 10 years that show some promise for restoring upper limb function of stroke survivors. Two of these methods are robotics and functional electrical stimulation (FES). These two therapeutic modalities are promising in that each has the capability to address particular impairments exhibited by stroke survivors, including weakness, hypertonia or spasticity, and dyscoordination, all of which may underlie upper limb dysfunction.

#### *Response to Functional Electrical Stimulation (FES)*

Response to FES has been studied for decades. Some early cohort studies demonstrated the feasibility and promise of using FES in chronic (>6 months) stroke survivors to improve the impairments of weakness, dyscoordination, and spasticity. Four early RCTs reported a statistically significant advantage in the use of FES for improving muscle strength vs. other available treatments for wrist extensors[63], knee extensors[49], ankle dorsiflexion[64], and wrist and finger flexors and extensors[65]. Glanz et al.[66] performed a meta-analysis study of FES for those studies published from 1978 to 1992, ultimately identifying the above four studies as RCTs and qualifying for meta-analysis with a common measurement metric, muscle force. According to meta-analysis, they found a good effect size of 0.63 for the four combined studies for FES-induced muscle force gains vs. other available interventions (95% CI 0.29–0.98; $p < 0.05$).

There is evidence that FES can reduce upper limb spasticity. In cohort studies of chronic stroke survivors with some residual elbow extension, FES intervention showed statistically significant reduction of abnormal biceps activation (EMG amplitude) during an elbow extension task[67,68], and either mean improvement or statistically significant improvement in Ashworth spasticity measures of shoulder, elbow, wrist, and finger muscles([69,70], respectively).

Researchers showed that FES was promising as a means to target dyscoordination. Some have used the Fugl-Meyer Coordination measure (FM) and found improvement in cohort study design (FM grasp item[71]) as well as an RCT that demonstrated a statistically significant advantage of FES, according to FM[72].

As in the previously described studies, it is important to target and measure treatment response according to upper limb impairments, such as weakness, spasticity, and dyscoordination, but it is critical that interventions result in sufficient impairment gain to produce measured functional gains. Activity-dependent CNS plasticity and principles of motor learning can serve to guide the generation of an intervention that not only improves impairment, but also effects significant functional gains. Two critical principles are training specificity and close attention to the motor practice. In order to provide these conditions, some researchers developed a motor learning protocol[73] composed of an array of tasks and task components. However, a volitionally practiced motor task can be practiced only for those who can at least partially perform the task. Many stroke survivors are unable. More recently, several researchers provided evidence that FES, when combined in a productive manner with other available methods, can produce functional gains. FES was capable of inducing muscle contractions and joint movements that were otherwise not possible to practice. For example, the surface FES could extend the wrist and fingers in preparation for grasping an object. In a cohort study, Alon and colleagues[70] described a method of combining FES for wrist and hand muscles with functional movement practice. In mild to moderately involved stroke survivors, they showed that this combination treatment resulted in statistically significant pre-/post-treatment gains in standardized measures of functional movements, such as grasp and release (Jebsen Heavy Cylinder and Light Cylinder subscales), and translational shoulder movements in the horizontal plane (Box and Blocks test[70]). In two recent RCTs, two other groups showed that there is a statistically significant advantage for FES combined with functional task component practice vs. the same treatment without FES. These results were shown with measures of actual functional tasks. For example, Popovich et al.[74] studied those who were mildly involved (<6 months) and they used the Upper Extremity Functional Test[74]. Daly and colleagues[75] studied those who were severely involved, and found a statistically significant advantage for combined FES (wrist/finger muscles) plus functional practice vs. functional practice and an alternative intervention, according to a subscale of the Arm Motor Abilities Test, a measure of wrist/hand functional task movements[75].

### *Response to Robotics Training*

Robotics applications for stroke survivors have a shorter history compared to FES. A number of investigators have shown that robotics can improve active shoulder movement[76], strengthening, and coordination[77]. An RCT showed that there was a statistically significant advantage of robotics vs. conventional exercise, according to shoulder/elbow coordination[78]. Scientists have successfully utilized robotics technology to elucidate important aspects of motor recovery, such as muscle activation patterns[80], smooth movement control[82], and the use of motor challenge vs. movement assistance[83]. More recently, others substantiated gains in impairment in response to robotics[79,80,81], but no assessment of accompanying functional gain was provided in response to robotics, or there was not significant function gain in the measures used (e.g., the FIM[78]).

### Measurement Development

In testing upper limb functional response to rehabilitation, it is important to avoid the measurement problems described above, such as subjectivity of self-report (Motor Activity Log (MAL)[37,84]), heterogeneous measures[37], and absence of measures of everyday functional tasks.

During the development of new intervention methods, it is sometimes necessary to limit interventions to motor control of a given joint(s). In that case, it is critical to utilize an outcome measure that is constructed to focus on only the motor control of joint movement(s) treated. To meet the need for focused, functional task assessment of either forearm/wrist/hand motor control or shoulder/elbow motor control, we used two strategies. First, we identified individual items within an existing impairment measure that could differentiate treatment response at an individual joint. For example, an individual item in the Fugl-Meyer Coordination scale captured the coordination ability of the finger extensors that was needed to release an object from the hand after completion of a task[75]. Second, we constructed two separate functional task measures. These were limited to either forearm/wrist/hand or elbow/shoulder functional task components. The functional task measures were based on the existing Arm Motor Ability Test (AMAT[85]), composed of 13 complex tasks (e.g., cut the meat with knife and fork). We devised a method to measure gains in the actual complex shoulder/elbow movements (AMAT S/E) or forearm/wrist/hand movements (AMAT W/H[75]) that composed the 13 functional tasks. We tested the performance of the measures, finding them valid, reliable, and capable of discriminating treatment response[75].

### Results of Implementing an Evidence-Based Intervention with Model-Based Measures

In a small RCT (n =12 stroke survivors; >12 months after stroke), we tested a combination of shoulder/elbow robotics and motor learning (ROB ML) vs. a combination of wrist/hand FES and motor learning (FES ML) in severely involved chronic stroke survivors. These two combination protocols were developed to incorporate important motor learning principles associated with CNS plasticity. Both robotics and FES provided different and overlapping elements of coordination training required by the motor learning principles associated with the CNS plasticity model. Table 2 lists the practice advantages of robotics, FES, and motor learning without therapeutic agents. For the robotics application, the subject was in a seated position and practiced linear movements in eight different directions on a horizontal work surface with the forearm in a supporting cradle. For the FES application, subjects were in a seated position at a work station, and FES was applied to the wrist and finger flexors and extensors. When FES activated wrist extensors and finger flexors, the subject practiced grasping an object. When FES activated wrist extensors and finger extensors, the subject practiced releasing the object. FES stimulation parameters were as follows: 50 Hz, 300-μsec pulse width, and amplitude within comfort of the subject.

We found that ROB ML produced a statistically significant gain in AMAT S/E, whereas FES ML did not. We found that FES ML produced a statistically significant gain in AMAT W/H, whereas ROB ML did not[70]. These results together support the phenomenon of training specificity in that the most practiced joint movements improved in comparison to joint movements that were practiced at a lesser frequency. It is possible that this study was successful in producing gains in a functional measure because the intervention was planned in accordance with principles of motor learning–associated motor skill acquisition, according to CNS plasticity. A second potential reason for successful demonstration of

**TABLE 2**
**Practice Advantages of Motor Learning, Robotics, and FES**

| | Motor Learning | Robotics | FES* |
|---|---|---|---|
| Passive movement, automatic | | Yes | |
| Supported limb | Yes | Yes | Yes |
| Unsupported limb | Yes | Depending on the robot model | Yes |
| Muscle activated, automatic | | | Yes |
| Automatically induced movement from muscle activation | | | Yes |
| Scapular stability training | Yes | | Yes |
| Multijoint movement practice | Yes | Restricted to robot capability | Yes |
| Whole limb movement practice | Yes | Limited to robot capability | Yes |
| Movement components of functional tasks | Yes | Restricted to robot capability | Yes |
| Functional task practice | Yes | | Yes |
| Visual feedback for fine control of movement trajectory maintenance, target accuracy, and movement smoothness | | Yes | |

*For some (<20%), sensation of FES is not comfortable at therapeutic levels (Rogers J, Daly J. 2006. Clinical Research Records, Stroke Motor Control and Motor Learning Laboratory, Research Service, Louis Stokes Cleveland Department of Veterans Affairs, Cleveland, Ohio.)

functional gain in response to targeted treatment was that the measures were designed for accuracy and sensitivity in measuring the response to each specific intervention.

### RELATIONSHIP BETWEEN BRAIN PLASTICITY MOTOR LEARNING PRINCIPLES AND THE ICF HEALTH SYSTEMS MODEL

If we are willing to adhere to the ICF model, we accept the challenge that the goal of rehabilitation is life role participation, with functional improvement as in important intermediary step. The ICF model suggests that we intervene at multiple lower levels (e.g., pathology and impairment) in order to improve the higher levels of function and life role participation. The ICF model also suggests that we measure at each level. Not only can we then understand response to treatment at each level, but we can also begin to understand relationships between levels (e.g., impairment and function).

With the ICF model proffering the challenge of restoring life role participation, it then becomes important to design and test interventions that result in impairment gains sufficiently robust to be reflected in functional activities and, further, in life role participation. Fortunately, CNS plasticity and associated motor learning principles can serve well as the basis for generating such interventions. These principles were useful in generating both efficacious gait training and upper limb functional training interventions. These principles led to the use of therapeutic agents (FES and robotics) so that close-to-normal movements could be practiced. These principles supported the use of specific therapeutic agents (BWSTT, FES, and robotics) so that sufficient movement repetition could be provided. These principles also supported incorporation of functional task practice and the demand of attention to task practice within the intervention.

The ICF model provided the challenge to restore function and life role participation. The means to that end were provided by CNS plasticity and associated principles of motor learning.

### ACKNOWLEDGEMENT

Department of Veterans Affairs, Rehabilitation Research and Development, Grants B2226R, A3102R.

### REFERENCES

1. Verbrugge, L.M. and Jette, A.M. (1994) The disablement process. *Soc. Sci. Med*. **38,** 1–14.
2. Jette, A.M. (1994) Physical disablement concepts for physical therapy research and practice. *Phys. Ther.* **74,** 380–386.
3. WHO (2001) International Classification of Functioning, Disability and Health. World Health Organization, Geneva.
4. Kolb, B. and Gibb, R. (1991) Environmental enrichment and cortical injury: behavioral and anatomical consequences of frontal cortex lesions. *Cereb. Cortex* **1,** 189–198.
5. Foster, T.C. and Dumas, T.C. (2001) Mechanism for increased hippocampal synaptic strength following differential experience. *J. Neurophysiol*. **85,** 1377–1383.
6. Kempermann, G., Kuhn, H.G., and Gage, F.H. (1997) Genetic influence on neurogenesis in the dentate gyrus of adult mice. *Proc. Natl. Acad. Sci. U. S. A.* **94,** 10409–10414.
7. Kleim, J.A., Lussnig, E., Schwarz, E.R., Comery, T.A., and Greenough, W.T. (1996) Synaptogenesis and Fos expression in the motor cortex of the adult rat after motor skill learning. *J. Neurosci.* **16,** 4529–4535.
8. Nudo, R.J., Wise, B.M., SiFuentes, F., and Milliken, G.W. (1996) Neural substrates for the effects of rehabilitative training on motor recovery after ischemic infarct. *Science* **272,** 1791–1794.
9. Nudo, R.J., Milliken, G.W., Jenkins, W.M., and Merzenich, M.M. (1996) Use-dependent alterations of movement representations in primary motor cortex of adult squirrel monkeys. *J. Neurosci.* **16,** 785–807.
10. Biernaskie, J. and Corbett, D. (2001) Enriched rehabilitative training promotes improved forelimb motor function and enhanced dendritic growth after focal ischemic injury. *J. Neurosci.* **21,** 5272–5280.
11. Jones, T.A., Chu, C.J., Grande, L.A., and Gregory, A.D. (1999) Motor skills training enhances lesion-induced structural plasticity in the motor cortex of adult rats. *J. Neurosci.* **19,** 10153–10163.
12. Chu, C.J. and Jones, T.A. (2000) Experience-dependent structural plasticity in cortex heterotopic to focal sensorimotor cortical damage. *Exp. Neurol.* **166,** 403–414.
13. Nelles, G., Jentzen, W., Jueptner, M., Muller, S., and Diener, H.C. (2001) Arm training induced brain plasticity in stroke studied with serial positron emission tomography. *Neuroimage* **13,** 1146–1154.
14. Ward, N.S., Brown, M.M., Thompson, A.J., and Frackowiak, R.S. (2003) Neural correlates of outcome after stroke: a cross-sectional fMRI study. *Brain* **126,** 1430–1448.

15. Neumann-Haefelin, T., Moseley, M.E., and Albers, G.W. (2000) New magnetic resonance imaging methods for cerebrovascular disease: emerging clinical applications. *Ann. Neurol.***47,** 559–570.
16. Newton, J., Sunderland, A., Butterworth, S.E., Peters, A.M., Peck, K.K., and Gowland, P.A. (2002) A pilot study of event-related functional magnetic resonance imaging of monitored wrist movements in patients with partial recovery. *Stroke* **33,** 2881–2887.
17. Liepert, J., Miltner, W.H., Bauder, H., Sommer, M., Dettmers, C., Taub, E., and Weiller, C. (1998) Motor cortex plasticity during constraint-induced movement therapy in stroke patients. *Neurosci. Lett.* **250,** 5–8.
18. Liepert, J., Uhde, I., Graf, S., Leidner, O., and Weiller, C. (2001) Motor cortex plasticity during forced-use therapy in stroke patients: a preliminary study. *J. Neurol.* **248,** 315–321.
19. Marshall, R.S., Perera, G.M., Lazar, R.M., Krakauer, J.W., Constantine, R.C., and DeLaPaz, R.L. (2000) Evolution of cortical activation during recovery from corticospinal tract infarction. *Stroke* **31,** 656–661.
20. Carey, J.R., Kimberley, T.J., Lewis, S.M., Auerbach, E.J., Dorsey, L., Rundquist, P., and Ugurbil, K. (2002) Analysis of fMRI and finger tracking training in subjects with chronic stroke. *Brain* **125,** 773–788.
21. Johansen-Berg, H., Dawes, H., Guy, C., Smith, S.M., Wade, D.T., and Matthews, P.M. (2002) Correlation between motor improvements and altered fMRI activity after rehabilitative therapy. *Brain* **125,** 2731–2742.
22. Umphred, D. (1995) *Neurological Rehabilitation.* Mosby.
23. Carr, J. and Shepard, R. (1987) A motor learning model for rehabilitation. In *Movement Science: Foundations for Physical Therapy in Rehabilitation.* Carr, J. and Shepard, R., Eds. Aspen Publications, Rockville, MD.
24. Gordon, J. (1987) Assumptions underlying physical therapy intervention: theoretical and historical perspectives. In *Movement Science: Foundations for Physical Therapy in Rehabilitation.* Carr, J. and Shepard, R., Eds. Aspen Publications, Rockville, MD.
25. Gubbay, S.S. (1978) The management of developmental apraxia. *Dev. Med. Child Neurol.* **20,** 643–646.
26. Singer, R., Lidor, R., and Cauraugh, J.H. (1993) To be aware or not aware? What to think about while learning and performing a motor skill. *Sport Psychol.* **7,** 19–30.
27. Butefisch, C., Hummelsheim, H., Denzler, P., and Mauritz, K.H. (1995) Repetitive training of isolated movements improves the outcome of motor rehabilitation of the centrally paretic hand. *J. Neurol. Sci.* **130,** 59–68.
28. Dean, C.M. and Shepherd, R.B. (1997) Task-related training improves performance of seated reaching tasks after stroke. A randomized controlled trial. *Stroke* **28,** 722–728.
29. Elbert, T., Pantev, C., Wienbruch, C., Rockstroh, B., and Taub, E. (1995) Increased cortical representation of the fingers of the left hand in string players. *Science* **270,** 305–307.
30. Pascual-Leone, A. and Torres, F. (1993) Plasticity of the sensorimotor cortex representation of the reading finger in Braille readers. *Brain* **116(Pt 1),** 39–52.
31. Plautz, E.J., Milliken, G.W., and Nudo, R.J. (2000) Effects of repetitive motor training on movement representations in adult squirrel monkeys: role of use versus learning. *Neurobiol. Learn. Mem.* **74,** 27–55.
32. Lawrence, R.H. and Jette, A.M. (1996) Disentangling the disablement process. *J. Gerontol. B Psychol. Sci. Soc. Sci.* **51,** S173–182.
33. Duncan, P.W., Lai, S.M., and Keighley, J. (2000) Defining post-stroke recovery: implications for design and interpretation of drug trials. *Neuropharmacology* 39, 835–841.
34. Granger, C.V. and Hamilton, B.B. (1990) Measurement of stroke rehabilitation outcome in the 1980s. *Stroke* **21,** II46–47.
35. Hamilton, B.B., Granger, C.V., and Sherwin, F. (1987) A uniform national data system for medical rehabilitation. In *Rehabilitation Outcomes: Analysis and Measurement.* Fuhrer, M.J., Ed. Paul H. Brookes, Baltimore.
36. Whiteneck, G.G., Charlifue, S.W., Gerhart, K.A., Overholser, J.D., and Richardson, G.N. (1992) Quantifying handicap: a new measure of long-term rehabilitation outcomes. *Arch. Phys. Med. Rehabil.* **73,** 519–526.
37. Wolf, S.L., Catlin, P.A., Ellis, M., Archer, A.L., Morgan, B., and Piacentino, A. (2001) Assessing Wolf motor function test as outcome measure for research in patients after stroke. *Stroke* **32,** 1635–1639.
38. Tinetti, M.E. (1986) Performance-oriented assessment of mobility problems in elderly patients. *J. Am. Geriatr. Soc.* **34,** 119–126.
39. Daly, J.J. and Ruff, R.L. (2004) Feasibility of combining multi-channel functional neuromuscular stimulation with weight-supported treadmill training. *J. Neurol. Sci*. **225,** 105–115.
40. Hesse, S., Helm, B., Krajnik, J., Gregoric, M., and Mauritz, K.H. (1997) Treadmill training with partial body weight support: influence of body weight release on the gait of hemiparetic patients. *Neurorehabil. Neural Repair* **11,** 15–20.
41. Visintin, M. and Barbeau, H. (1994) The effects of parallel bars, body weight support and speed on the modulation of the locomotor pattern of spastic paretic gait. A preliminary communication. *Paraplegia* **32,** 540–553.
42. Trueblood, P.R. (2001) Partial body weight treadmill training in persons with chronic stroke. *NeuroRehabilitation* **16,** 141–153.
43. Visintin, M., Barbeau, H., Korner-Bitensky, N., and Mayo, N.E. (1998) A new approach to retrain gait in stroke patients through body weight support and treadmill stimulation. *Stroke* **29,** 1122–1128.
44. Moseley, A.M., Stark, A., Cameron, I.D., and Pollock, A. (2005) Treadmill training and body weight support for walking after stroke. *Cochrane Database Syst. Rev.* CD002840.
45. Kwakkel, G. and Wagenaar, R.C. (2002) Effect of duration of upper- and lower-extremity rehabilitation sessions and walking speed on recovery of interlimb coordination in hemiplegic gait. *Phys. Ther.* **82,** 432–448.

46. Patten, C., Lexell, J., and Brown, H.E. (2004) Weakness and strength training in persons with poststroke hemiplegia: rationale, method, and efficacy. *J. Rehabil. Res. Dev.* **41,** 293–312.
47. Meesala, S. and Daly, J.J. (2004) Differential impact of lower limb strength and coordination on gait swing phase restoration following seven years of persistent gait deficits. *Archives of Phys Med and Rehabil*, **85(9)**, e35.
48. Waters, R.L., McNeal, D., and Perry, J. (1975) Experimental correction of footdrop by electrical stimulation of the peroneal nerve. *J. Bone Joint Surg. Am.* **57,** 1047–1054.
49. Winchester, P., Montgomery, J., Bowman, B., and Hislop, H. (1983) Effects of feedback stimulation training and cyclical electrical stimulation on knee extension in hemiparetic patients. *Phys. Ther.* **63,** 1096–1103.
50. Daly, J.J. and Ruff, R.L. (2000) Electrically induced recovery of gait components for older patients with chronic stroke. *Am. J. Phys. Med. Rehabil.* **79,** 349–360.
51. Daly, J.J., Barnickle, K., Kobetic, R., and Marsolais, E.B. (1993) Electrically induced gait changes post stroke, using an FNS system with intramuscular electrodes and multiple channels. *J. Neurol. Rehabil.* 17–25.
52. McNeal, D. and Reswick, J.B. (1976) Control of skeletal muscle by electrical stimulation. *Adv. Biomed. Eng.* **6,** 209–256.
53. Bogataj, U., Gros, N., Kljajic, M., Acimovic, R., and Malezic, M. (1995) The rehabilitation of gait in patients with hemiplegia: a comparison between conventional therapy and multichannel functional electrical stimulation therapy. *Phys. Ther*. **75,** 490–502.
54. Daly, J.J., Kollar, K., Debogorski, A.A., Strasshofer, B., Marsolais, E.B., Scheiner, A., Snyder, S., and Ruff, R.L. (2001) Performance of an intramuscular electrode during functional neuromuscular stimulation for gait training post stroke. *J. Rehabil. Res. Dev.* **38,** 513–526.
55. Daly, J.J., Ruff, R.L., Osman, S., and Hull, J.J. (2000) Response of prolonged flaccid paralysis to FNS rehabilitation techniques. *Disabil. Rehabil.* **22,** 565–573.
56. Moseley, A.M., Wales, A., Herbert, R., Schurr, K., and Moore, S. (1993) Observation and analysis of hemiplegic gait: stance phase. *Aust. J. Physiother*. **39,** 259–267.
57. Moore, S., Schurr, K., Wales, A., Moseley, A., and Herbert, R. (1993) Observation and analysis of hemiplegic gait: swing phase. *Aust. J. Physiother.* **39,** 271–278.
58. Kerrigan, D.C., Gronley, J., and Perry, J. (1991) Stiff-legged gait in spastic paresis. A study of quadriceps and hamstrings muscle activity. *Am. J. Phys. Med. Rehabil.* **70,** 294–300.
59. Daly, J.J., Roenigk, K., Holcomb, J., Rogers, J.M., Butler, K., Gansen, J., McCabe, J., Fredrickson, E., Marsolais, E.B., and Ruff, R.L. (2006) A randomized controlled trial of functional neuromuscular stimulation in chronic stroke subjects. *Stroke* **37,** 172–178.
60. Daly, J.J., Sng, K., Roenigk, K., Fredrickson, E., and Dohring, M. (2006) Intra-limb coordination deficit in stroke survivors and response to treatment. *Gait Posture.* **25(3),** 412–418.
61. Marsolais, E.B. and Kobetic, R. (1986) Implantation techniques and experience with percutaneous intermuscular electrodes in the lower extremities. *J. Rehabil. Res. Dev*. **23,** 1–8.
62. Daly, J.J., Ruff, R.L., Haycook, K., Strasshofer, B., Marsolais, E.B., and Dobos, L. (2000) Feasibility of gait training for acute stroke patients using FNS with implanted electrodes. *J. Neurol. Sci.* **179,** 103–107.
63. Bowman, B.R., Baker, L.L., and Waters, R.L. (1979) Positional feedback and electrical stimulation: an automated treatment for the hemiplegic wrist. *Arch. Phys. Med. Rehabil.* **60,** 497–502.
64. Merletti, R., Zelaschi, F., Latella, D., Galli, M., Angeli, S., and Sessa, M.B. (1978) A control study of muscle force recovery in hemiparetic patients during treatment with functional electrical stimulation. *Scand. J. Rehabil. Med.* **10,** 147–154.
65. Powell, J., Pandyan, A.D., Granat, M., Cameron, M., and Stott, D.J. (1999) Electrical stimulation of wrist extensors in poststroke hemiplegia. *Stroke* **30,** 1384–1389.
66. Glanz, M., Klawansky, S., Stason, W., Berkey, C., and Chalmers, T.C. (1996) Functional electrostimulation in poststroke rehabilitation: a meta-analysis of the randomized controlled trials. *Arch. Phys. Med. Rehabil.* **77,** 549–553.
67. Lagasse, P.P. and Roy, M.A. (1989) Functional electrical stimulation and the reduction of co-contraction in spastic biceps brachii. *Clin. Rehabil.* **31,** 11–116.
68. Dimitrijevic, M.M., Stokic, D.S., Wawro, A.W., and Wun, C.C. (1996) Modification of motor control of wrist extension by mesh-glove electrical afferent stimulation in stroke patients. *Arch. Phys. Med. Rehabil.* **77,** 252–258.
69. Hummelsheim, H., Maier-Loth, M.L., and Eickhof, C. (1997) The functional value of electrical muscle stimulation for the rehabilitation of the hand in stroke patients. *Scand. J. Rehabil. Med.* **29,** 3–10.
70. Alon, G., Sunnerhagen, K.S., Geurts, A.C., and Ohry, A. (2003) A home-based, self-administered stimulation program to improve selected hand functions of chronic stroke. *NeuroRehabilitation* **18,** 215–225.
71. Alon, G., McBride, S.K., and Ring, H. (2002) Improving selected hand functions using upper extremity noninvasive neuroprosthesis in persons with chronic stroke. *J. Stroke Cerebrovasc. Dis.* **11,** 99–106.
72. Chae, J., Bethoux, F., Bohine, T., Dobos, L., Davis, T., and Friedl, A. (1998) Neuromuscular stimulation for upper extremity motor and functional recovery in acute hemiplegia. *Stroke* **29,** 975–979.
73. Dromerick, A.W., Lum, P.S., and Hidler, J. (2006) Activity-based therapies. *NeuroRx* **3,** 428–438.
74. Popovic, M.B., Popovic, D.B., Sinkjaer, T., Stefanovic, A., and Schwirtlich, L. (2003) Clinical evaluation of Functional Electrical Therapy in acute hemiplegic subjects. *J. Rehabil. Res. Dev.* **40,** 443–453.
75. Daly, J.J., Hogan, N., Perepezko, E.M., Krebs, H.I., Rogers, J.M., Goyal, K.S., Dohring, M.E., Fredrickson, E.,

Nethery, J., and Ruff, R.L. (2005) Response to upper-limb robotics and functional neuromuscular stimulation following stroke. *J. Rehabil. Res. Dev.* **42,** 723–736.

76. Kahn, L.E., Averbuch, M., Rymer, W.Z., and Reinkensmeyer, D.J. (2001) Comparison of robot-assisted reaching to free reaching in promoting recovery from chronic stroke. In *Integration of Assistive Technology in the Information Age.* Mokhari, M., Ed. IOS Press. pp. 39–44.
77. Fasoli, S.E., Krebs, H.I., Stein, J., Frontera, W.R., and Hogan, N. (2003) Effects of robotic therapy on motor impairment and recovery in chronic stroke. *Arch. Phys. Med. Rehabil.* **84,** 477–482.
78. Volpe, B.T., Krebs, H.I., and Hogan, N. (2001) Is robot-aided sensorimotor training in stroke rehabilitation a realistic option? *Curr. Opin. Neurol.* **14,** 745–752.
79. Hesse, S., Werner, C., Pohl, M., Rueckriem, S., Mehrholz, J., and Lingnau, M.L. (2005) Computerized arm training improves the motor control of the severely affected arm after stroke: a single-blinded randomized trial in two centers. *Stroke* **36,** 1960–1966.
80. Lum, P.S., Burgar, C.G., and Shor, P.C. (2004) Evidence for improved muscle activation patterns after retraining of reaching movements with the MIME robotic system in subjects with post-stroke hemiparesis. *IEEE Trans. Neural. Syst. Rehabil. Eng.* **12,** 186–194.
81. Fasoli, S.E., Krebs, H.I., Stein, J., Frontera, W.R., Hughes, R., and Hogan, N. (2004) Robotic therapy for chronic motor impairments after stroke: follow-up results. *Arch. Phys. Med. Rehabil.* **85,** 1106–1111.
82. Hogan, N. and Krebs, H.I. (2004) Interactive robots for neuro-rehabilitation. *Restor. Neurol. Neurosci.* **22,** 349–358.
83. Patton, J.L., Stoykov, M.E., Kovic, M., and Mussa-Ivaldi, F.A. (2006) Evaluation of robotic training forces that either enhance or reduce error in chronic hemiparetic stroke survivors. *Exp. Brain Res.* **168,** 368–383.
84. Taub, E., Miller, N.E., Novack, T.A., Cook, E.W., 3rd, Fleming, W.C., Nepomuceno, C.S., Connell, J.S., and Crago, J.E. (1993) Technique to improve chronic motor deficit after stroke. *Arch. Phys. Med. Rehabil.* **74,** 347–354.
85. Kopp, B., Kunkel, A., Flor, H., Platz, T., Rose, U., Mauritz, K.H., Gresser, K., McCulloch, K.L., and Taub, E. (1997) The Arm Motor Ability Test: reliability, validity, and sensitivity to change of an instrument for assessing disabilities in activities of daily living. *Arch. Phys. Med. Rehabil.* **78,** 615–620.

**This article should be cited as follows:**

Daly, J.J. and Ruff, R.L. (2007) Construction of efficacious gait and upper limb functional interventions based on brain plasticity evidence and model-based measures for stroke patients. *TheScientificWorldJOURNAL* **7**, 2031–2045. DOI 10.1100/tsw.2007.299.
